# Tackling the emperor’s wisdom: heat shock proteins to halt and reverse atrial fibrillation at its roots

**DOI:** 10.1007/s12471-015-0698-1

**Published:** 2015-05-14

**Authors:** B.C. du Pré, T.A.B. van Veen

**Affiliations:** Division of Heart and Lungs, Department of Medical Physiology, University Medical Center Utrecht, Utrecht, The Netherlands

‘When the pulse is irregular and tremulous and the beats occur at intervals, then the impulse of life fades’. More than 4000 years ago, the Yellow Emperor of China wrote this in his classics of internal medicine (Huang Ti Nei Ching Su Wen) [1], perhaps thereby describing atrial fibrillation (AF) for the first time. Currently, AF is the most common sustained arrhythmia in Western society. Its prevalence of 1 % is expected to double in the next decades, primarily due to ageing of the population, making it a growing public health burden with an unacceptably high impact on quality of life. The pathophysiology of AF embeds a high mode of complexity, and the intermingling mechanisms underlying AF are still incompletely understood. In short, AF consists of heterogeneous electromechanical remodelling of the atria in a concealed phase, which is followed by presentation due to an initiating trigger. General modes of treatment focus on two main aspects: (1) termination of the initiating trigger, either by anti-arrhythmic medication, cardioversion, or ablation therapy, and (2) prevention of complications of AF, such as the formation of occluding thrombi or a high ventricular rate, which further exacerbates the pathophysiological consequences. However, curative treatment of the electromechanical remodelling, the main cause of AF development, maintenance, and progression, is currently not possible. The major problem for accurate and effective treatment of remodelling is the fact that at the moment of the first presentation of AF, the atria are already electrically and structurally remodelled to an advanced pathological level. As lessons from ancient history are still pertinent in today’s society, the saying of the Yellow Emperor is unfortunately still daily practice: AF patients have a doubled mortality risk.

In the current issue of *Netherlands Heart Journal*, Lanters et al. [2] describe the outline of the just-started HALT&REVERSE project, which aims to stop and possibly even reverse the electromechanical remodelling that causes AF using heat shock proteins (HSPs). The HSPs form a family of cardioprotective proteins that are up-regulated during cellular stress and function as intracellular chaperones for other proteins. They were discovered in relation to heat shock stress (hence the name), but they are present during all kinds of stress, including atrial stress. Previous studies in patients showed that impaired HSP expression is associated with AF, and HSP induction in various animal models resulted in atrial electrophysiological changes and inhibition of AF [3]. The use of HSPs and their inducers in the HALT&REVERSE project is therefore a very interesting idea, which targets AF at its roots, potentially curing the pathological remodelled heart.

The overall aim of the HALT&REVERSE project corresponds with an increasing mind-shift in cardiovascular research. Instead of only focusing on treatment of symptoms and complications, counteracting development of the underlying disease to be able to cure/limit/prevent the disease substrate, sometimes even before the onset of symptoms, is gaining more attention. In heart failure patients with an intraventricular conduction delay, for example, cardiac resynchronisation therapy (via intervention using electrical devices) is successfully used to reverse initial cardiac remodelling caused by altered activation [4]. Another illustrative example is a study by Asimaki et al. [5], investigating a genetic mutation in desmosomal proteins, which classically leads to arrhythmogenic cardiomyopathy (also known as arrhythmogenic right ventricular cardiomyopathy), a disease associated with a high incidence of sudden arrhythmogenic death. When cardiac remodelling was pharmacologically suppressed at an early stage, heart failure no longer developed, thereby treating the disease before its fatal onset. In this case, unravelling of the mechanism that initiates the cardiac remodelling, in combination with a sophisticated large drug-screening assay, opened doors for future curative therapy.

Curative treatment of the remodelled substrate in AF will not only support the immediate (but often short-lasting) success that cardioversion or ablation therapy have, but it also has the potential to prevent and reverse AF development, maintenance, and progression on the longer term and thereby significantly reduce the AF burden for patients and society. Let’s hope that research on such curative therapeutics will finally add new ground-breaking milestones to medicine and become part of daily practice in AF treatment, so that the emperor’s wisdom, as expressed in his ancient documentations 4000 years ago, will truly become history.

## Funding

None.

## Conflict of interest

None declared.
